# Engineering host‑defense peptides enhanced by artificial intelligence and nano delivery systems to overcome biofilms and antimicrobial resistance

**DOI:** 10.1016/j.engmic.2026.100277

**Published:** 2026-05-15

**Authors:** Raman Krishnamoorthi, Muthuramalingam Kaviyadharshini, Pambayan Ulagan Mahalingam, Moovendran Srinivash, Pitchaimuthu Rajkannan, Mohan Keerthivsan, Paulraj Suganya, Arokia Vijaya Anand Mariadoss

**Affiliations:** a Pharmaceutics Laboratory, Graduate Institute of Natural Products, Chang Gung University, Kweishan, Taoyuan, Taiwan; b Department of Biology, The Gandhigram Rural Institute (Deemed to be University) Gandhigram, Dindigul 624302, Tamil Nadu, India; c UPASI Tea Research Foundation, Reginal Centre, Coonoor, 643101 The Nilgiris, Tamil Nadu, India; d Biocon biologics limited, Electronic City, hosur road, Bangalore 560100, Karnataka, India; e Department of Biotechnology, Sri Kaliswari College (Autonomous), Sivakasi, Tamil Nadu 626123, India; f Department of Biological and Chemical Science, School of Liberal Arts and Sciences, Mohan Babu University, Tirupati 517102, Andhra Pradesh, India

**Keywords:** Antimicrobial peptides, Drug delivery, Bacterial infections

## Abstract

Infections caused by bacteria pose a risk to humanity as drugs become increasingly ineffective as resistance to bacterial strains emerge along with biofilm and persister formation. This review critically evaluates host defense peptides, rational design strategies that have guided next-generation antimicrobial peptide (AMP) discovery, and their current limitations. We also highlight optimization approaches including sequence engineering and chemical modification, synergistic combinations of antibiotics or adjuvants, and nanoscale delivery platforms that enhance stability, targeted delivery, and biofilm penetration. We also discuss the key chemical properties, delivery kinetics, and stimuli-responsive drug delivery for antibacterial and antibiofilm actions as well as the toxic effects of organic- and inorganic-based AMP delivery platforms. This underlines the importance of diverse modification techniques and artificial intelligence (AI)-assisted designs to improve the antibacterial activity, stability, and biocompatibility of AMPs. This study examines the latest advances in the combination of AMPs with drug delivery systems to improve clinical outcomes. Finally, the review discusses the clinical status, research gaps, current obstacles, and prospects of AMPs in antimicrobial resistance (AMR) therapy, offering key findings for the development of innovative AMPs with significant antibacterial activity, stability, and safety for AMR treatment.

## Highlights

• We discuss rational design and AI-guided strategies shaping next-gen AMP discovery.
• We highlight nano-delivery tactics improving AMP stability, targeting, and biofilm control.
• We critically reviewed synergistic AMP approaches enhancing efficacy and biocompatibility.
• Clinical gaps, obstacles, and prospects for AMPs in advancing AMR therapies are also discussed.

## Graphical abstract

**Figure:**
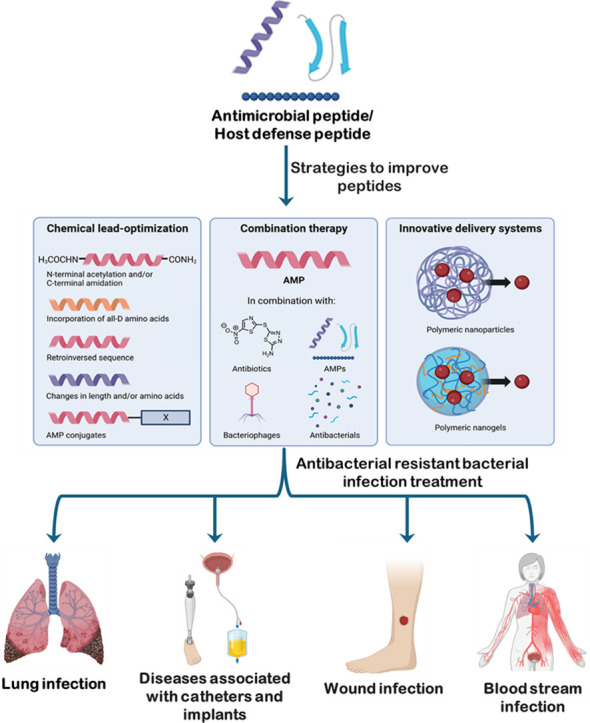

## 1 Introduction

In 1928, Alexander Fleming revolutionized the medical world with the discovery of penicillin [1]. Initially, Fleming’s discovery received little attention. However, in the 1940s, penicillin was produced in large quantities because of the efforts of Chain and Florey. As a result, penicillin entered the military scene at the end of World War II [2]. By the 1950s, a wide variety of antibiotic classes with different modes of action were discovered [3,4]. Antibiotics have since become one of the greatest medical advances in the 20th century for the treatment and prevention of bacterial infections, thereby saving millions of lives. Currently, advanced medical care depends heavily on the use of these antibiotics during transplantation and surgery. However, bacteria and other pathogens have evolved in response to the (ab)use of these antibiotics, resulting in the selection of antimicrobial-resistant strains [5]. In 1945, Fleming noted the danger of developing antimicrobial resistance (AMR) during his Nobel lecture, stating “It is not difficult to make microbes resistant to penicillin in the laboratory by exposing them to concentrations not sufficient to kill them, and the same thing has occasionally happened in the body” [1]. Bacteria can acquire resistance to antibiotics through the mutation of genes encoding enzymes, efflux pumps, and/or processes altering their cell wall, in effect selecting for optimal characteristics to remove the antibiotic from their system or through genetic exchange mechanisms with other bacteria [6]. AMR is one of the greatest public health threats faced by the global community, as it may lead to a scenario where simple infections, such as skin wounds or bladder infections, can no longer be treated with modern antibiotics [7]. Currently, approximately 50,000 deaths are caused by AMR annually in the United States and Europe; by 2050, this number is estimated to increase to 10 million people globally [8]. AMR strains of *Acinetobacter baumannii, Pseudomonas aeruginosa, Escherichia coli, Klebsiella pneumoniae*, and *Staphylococcus aureus* are increasingly encountered, constituting a major threat to public health [9].

**Fig. 1:**
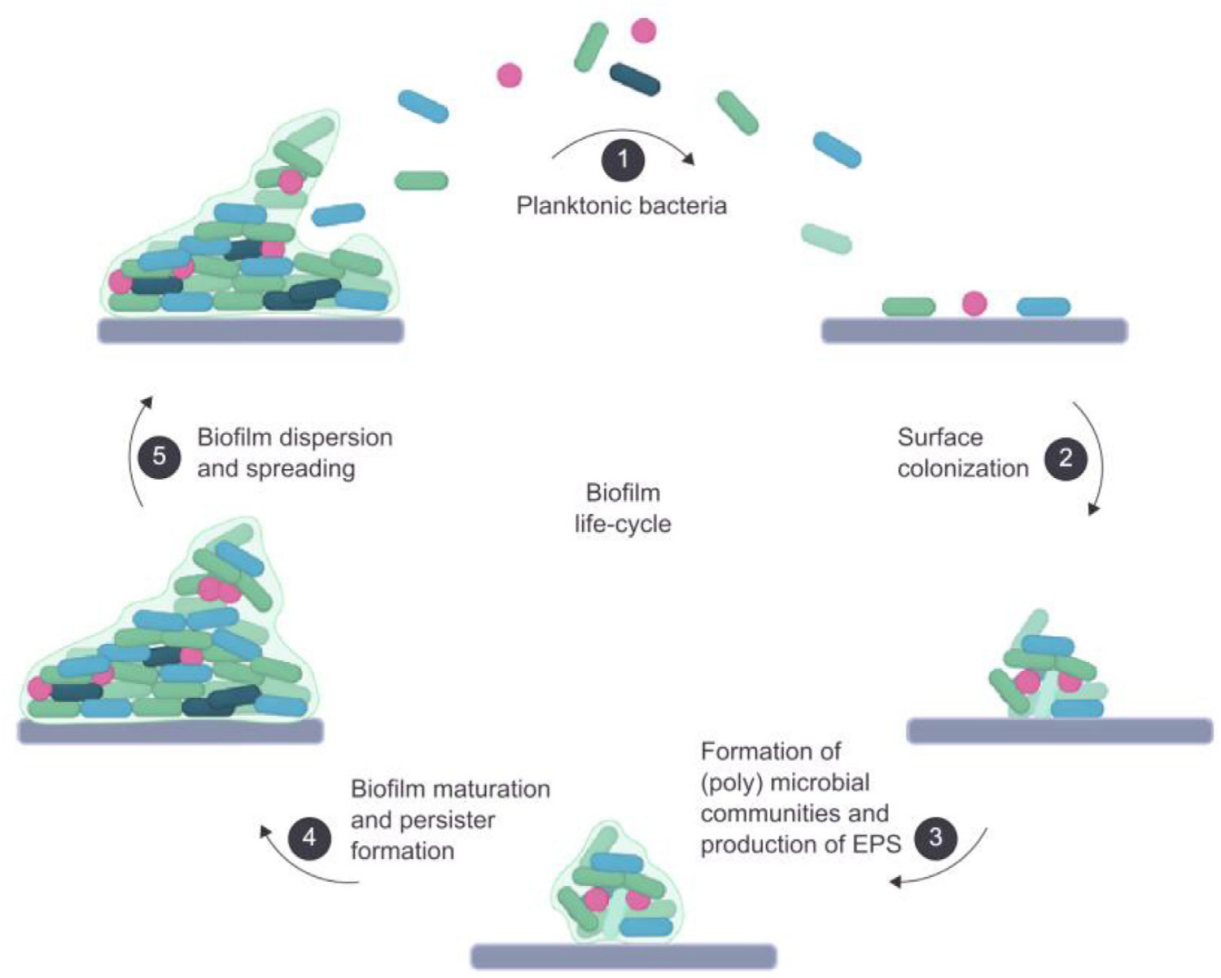
The steps involved in bacterial biofilm formation. Free floating planktonic bacteria (1) bind irreversibly to a surface, *e.g.* human skin or medical devices (2). These bacteria start to form (poly)microbial communities and produce extracellular polymeric substances (EPS) that form a biofilm matrix around the bacteria (3). As the biofilm develops and matures, some bacteria residing deeper inside the biofilm may change to a metabolically inactive state, into so-called persister cells (4). Over time, bacteria can disperse out of the biofilm, revert to a planktonic state, and start to colonize new surfaces (5). Figure is based on Sahli et al. [17]. The image was made using BioRender.

Biofilm and/or persister cell formation by these pathogens hamper the efficacy of antibiotics. Approximately 80% of chronic and recurrent bacterial infections are associated with biofilms [10]. Biofilms protect bacteria from environmental stressors such as antibiotics and effectors of the immune system, and are breeding grounds for the development of AMR [9,10]. Fig. 1 presents an overview of the different stages of biofilm formation. Biofilm formation is initiated after the irreversible attachment of planktonic bacterial cells to a surface, which could be human skin or medical devices such as catheters or implant materials [11]. After attachment, biofilms develop as (poly)microbial communities embedded in a self-produced extracellular matrix composed of polysaccharides, proteins, and DNA that protect the bacteria from hostile factors [12]. Within deeper layers of the biofilm, bacterial cells may change to a metabolically inactive state, the so-called persister cells [13]. These persister cells are more resistant to antibiotics that have bacterial targets involved in metabolism and have the capability to actively remove antibiotics from their system via efflux pumps [14]. Additionally, bacteria can hide inside host cells to evade the immune system and/or the effect of antibiotics [15]. The increasing failure of antibiotics and the lack of new antibiotics with a mode of action different from that of current antibiotics highlight the need for novel therapeutic agents.

Antimicrobial peptides (AMPs) are highly effective antibacterial agents. However, their nature and potential toxicity limit their use. This review critically evaluates host defense peptides, rational design strategies that have guided next-generation AMP discovery, and their current limitations. We also highlight optimization approaches, including sequence engineering and chemical modification, synergistic combinations of antibiotics or adjuvants, and nanoscale delivery platforms that enhance stability, targeted delivery, and biofilm penetration. We also discuss the key chemical properties, delivery kinetics, and stimulus-responsive drug delivery for antibacterial and antibiofilm actions as well as the toxic effects of organic- and inorganic-based AMP delivery platforms. We also aimed to determine the most cost-effective delivery mechanism and coating for AMP distribution to combat and prevent diseases that are difficult to treat. Furthermore, biophysical alterations, including peptide folding and structure, as well as *in silico* modeling and screening using artificial intelligence (AI), are now recognized as effective techniques for improving AMP. AI enables the rapid identification and development of antimicrobial peptide sequences as well as the prediction and optimization of peptide stability and activity [16]. This strategy improves the identification of novel AMPs, lowers R&D expenditures, and accelerates product development. These novel approaches provide several ways to increase the likelihood of AMPs acting as antimicrobial agents, overcome current obstacles, and open avenues for more promising infection-fighting drugs.

## 2 Antimicrobial (host defense) peptides

**Fig. 2:**
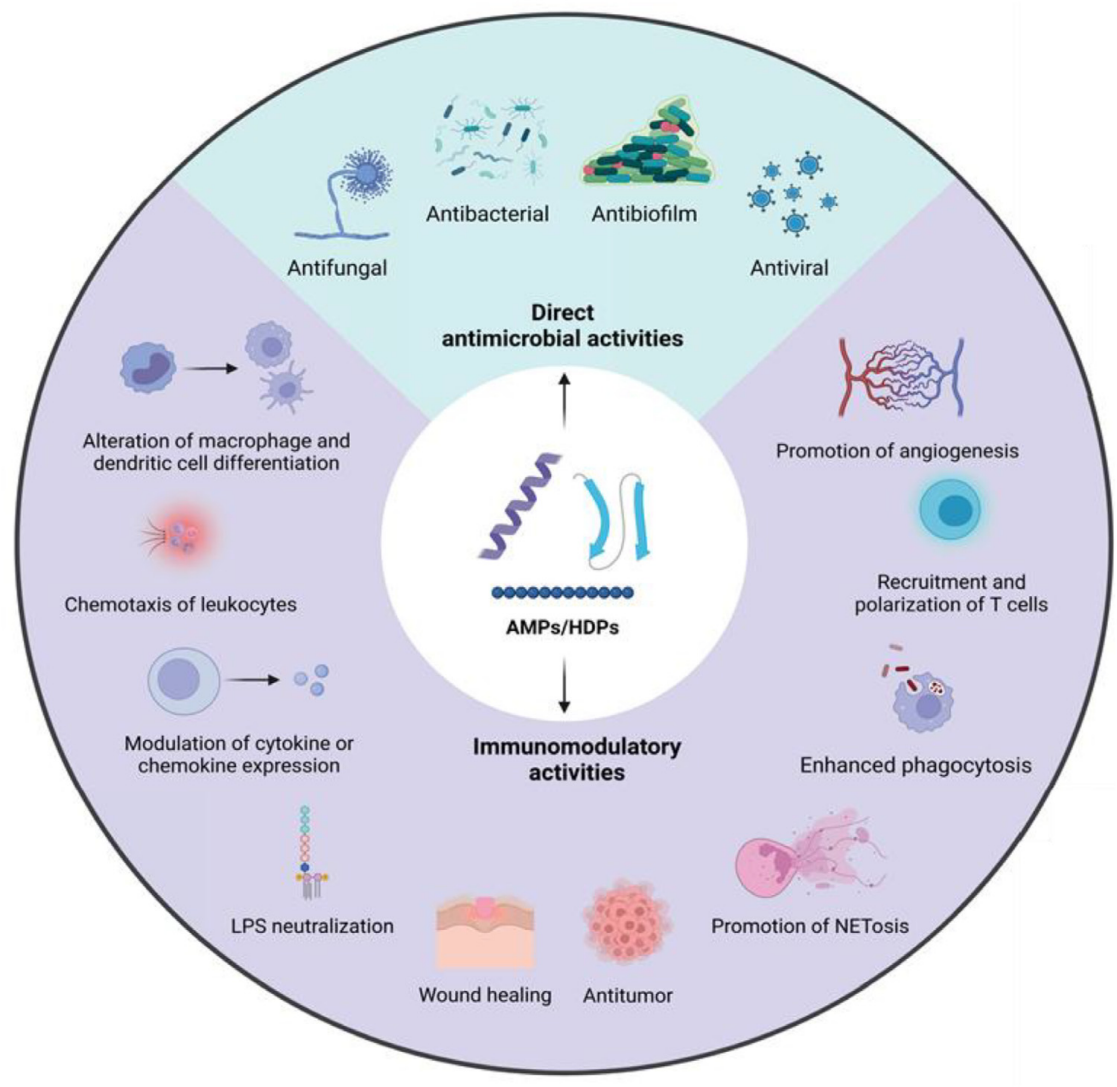
*Overview of the antimicrobial and immunomodulatory activities of antimicrobial peptides/host defense peptides.* The direct antimicrobial activities of these peptides include antifungal, antibacterial, and antiviral activities. The variety of their immunomodulatory activities range from effects on cells of the innate and adaptive immunity, as well as effects on tumors and wound environment. Figure is based on Di et al. [201]. The image was made using BioRender.

AMPs are a promising alternative to antibiotics [18]. AMPs are a class of small peptides that span 10–60 amino acids and are part of the innate immune response in a variety of organisms, including humans [19]. These peptides exhibit a wide range of biological activities, including antibacterial, antibiofilm, antiviral, antifungal, anticancer, wound-healing, and immunomodulatory activities [7,20,21]. To date, 3379 AMPs have been identified, and an APD6 Antimicrobial Peptide database (https://aps.unmc.edu/home) reporting potent AMPs originating from natural sources can be consulted. As AMPs are a diverse group of peptides, they can be classified based on various properties: i) source, ii) activity, iii) structure, and iv) amino acid-rich species (*e.g.* glycine, tryptophan, histidine, proline, and arginine) [22]. The best-studied structural classes of human AMPs are cathelicidins and defensins [23]. Two common characteristics of these AMPs are: they have a predominant cationic charge at physiological pH and contain ≥30% hydrophobic residues [24,25]. The cationic charge of these peptides drives the initial electrostatic interaction with the polyanionic outer surface of bacterial cells, that is, lipopolysaccharides or wall-associated teichoic acids in Gram-negative or Gram-positive bacteria, respectively [5,26]. AMPs are typically unstructured in aqueous solutions; however, in the presence of a biological membrane, they fold into an “active” amphipathic secondary structure, where hydrophobic residues are oriented opposite from cationic or polar residues [25]. Although the mechanisms of action of AMPs are diverse, direct interactions with, and disruption of, the bacterial membrane play key roles. Multiple destabilization models have been proposed to describe these interactions [20,27]. For instance, AMPs induce bacterial cell death by acting on intercellular targets after cellular internalization, which can affect DNA/RNA synthesis, protein synthesis, and protein folding [20]. Importantly, the risk of bacterial AMR development in response to AMPs is relatively low compared to conventional antibiotics, owing to their multifaceted mode of action [28]. It has long been thought that direct antimicrobial activities are the primary function of natural AMPs; however, recently, this consensus has changed, and immunomodulatory properties are now considered the primary role of natural AMPs [29,30]. Hence, their alternative name is host defense peptides (HDPs). The immunomodulatory properties of HDPs are diverse and include the following: i) chemotaxis of leukocytes, ii) alteration of macrophage and dendritic cell differentiation, and iii) modulation of cytokine and chemokine expression [29,30]. Furthermore, several HDPs stimulate angiogenesis and promote wound healing [31,32]. An overview of the direct antimicrobial and immunomodulatory activities of AMPs/HDPs is shown in Fig. 2.

## 3 Development of synthetic antimicrobial and antibiofilm peptide (SAAP)

The development of novel AMPs has been guided primarily by rational design principles and is increasingly being combined with computer-aided methods, such as artificial intelligence (AI), to improve structure-activity relationship predictions [33]. The only member of the human cathelicidin family is LL-37, which has moderate broad-spectrum antimicrobial activity [34] and is well known for its immunomodulatory activities [35,36]. LL-37 served as a starting point for the development of AMPs with improved bactericidal and antibiofilm activities [34]. First, a library of LL-37 derivatives, in which the core antimicrobial region was maintained, was synthesized, and the hit compound OP-145 (also named P60.4Ac) showed improved antimicrobial activity compared with LL-37 [37]. OP-145 was also effective in the prevention of *S. aureus* implant-associated infections in rabbits when incorporated into a biodegradable implant coating [38] or *S. aureus* infections in human epidermal models when incorporated into a hypromellose gel [39]. Moreover, OP-145 successfully cured chronic otitis media in 47% of cases in a phase II clinical trial [40]. Despite these successes, the antimicrobial activity of OP-145 was reduced in the presence of biological fluids such as human plasma, wound fluid, and urine [37]. Therefore, to further improve antimicrobial and antibiofilm activities under these physiological conditions, a second library of LL-37 derivatives was synthesized, which resulted in the lead peptide SAAP-148. SAAP-148 is a potent broad-spectrum agent that can eradicate multidrug-resistant bacteria in the ESKAPE panel (*Enterococcus faecium, S. aureus, K. pneumoniae, A. baumannii, P. aeruginosa*, and *Enterobacter* species) and can completely eradicate *S. aureus* and *A. baumannii* biofilm infections in murine skin [41]. Moreover, SAAP-148 can eradicate *S. aureus* persister cells in antibiotic-treated mature biofilms, simulating prosthetic joint infections [42]. SAAP-148 is also a membrane-disruptive AMP; its mode of action is ascribed to its insertion into the bacterial membrane, followed by membrane thinning, permeabilization, and leakage, resulting in bacterial cell death within minutes [41]. The improved antimicrobial activity of SAAP-148 is a result of its superior ability to disrupt the bacterial membrane compared to that of OP-145 [43]. Importantly, SAAP-148 showed almost no resistance development [41], indicating its therapeutic potential in the treatment of antimicrobial-resistant bacterial infections.

## 4 Limitations of AMPs and SAAP-148

To date, only seven AMPs (including lipo- and/or glycopeptides and their cyclic counterparts) have been translated to clinical applications: gramicidin, daptomycin, colistin, vancomycin, ritavancin, dalbavancin, and telavancin [44]. Due to their peptide nature, the pharmacokinetic and pharmacodynamic properties of AMPs have hampered their success in clinical trials [45]. Challenges for the further development of AMPs include: i) their relatively high hemolytic and cytotoxic activities at antimicrobial concentrations, resulting in low selectivity towards bacterial cells over mammalian cells [[46], [47], [48]]; ii) limited proteolytic stability due to peptide degradation by proteolytic enzymes produced by the host or bacteria [47,49]; iii) limited bioavailability due to binding to plasma and/or serum proteins [49,50]; iv) short systemic half-life [44]; v) limited penetration into tissue [49] or bacterial biofilms [51]; and vi) expensive production costs, especially for long peptide sequences [47,49]. In addition, SAAP-148 suffers from limitations related to its peptide nature, such as a small therapeutic window and short half-life owing to plasma protein binding [52,53]. Although SAAP-148 has proven successful in the treatment of superficial skin wound infections *in vivo* [41], its antibacterial activity has been limited to deeper skin wounds, such as surgical wound infections in rats [52]. Factors contributing to the reduced activity of SAAP-148 in this *in vivo* surgical wound infection model were related to i) components within the wound microenvironment (*e.g.*, proteins and proteases), ii) inadequate or relatively slow release from the hydrogel or wound dressing, and iii) re-colonization due to a relatively high bacterial load in the wounds [52].

## 5 Strategies to improve AMPs

**Fig. 3:**
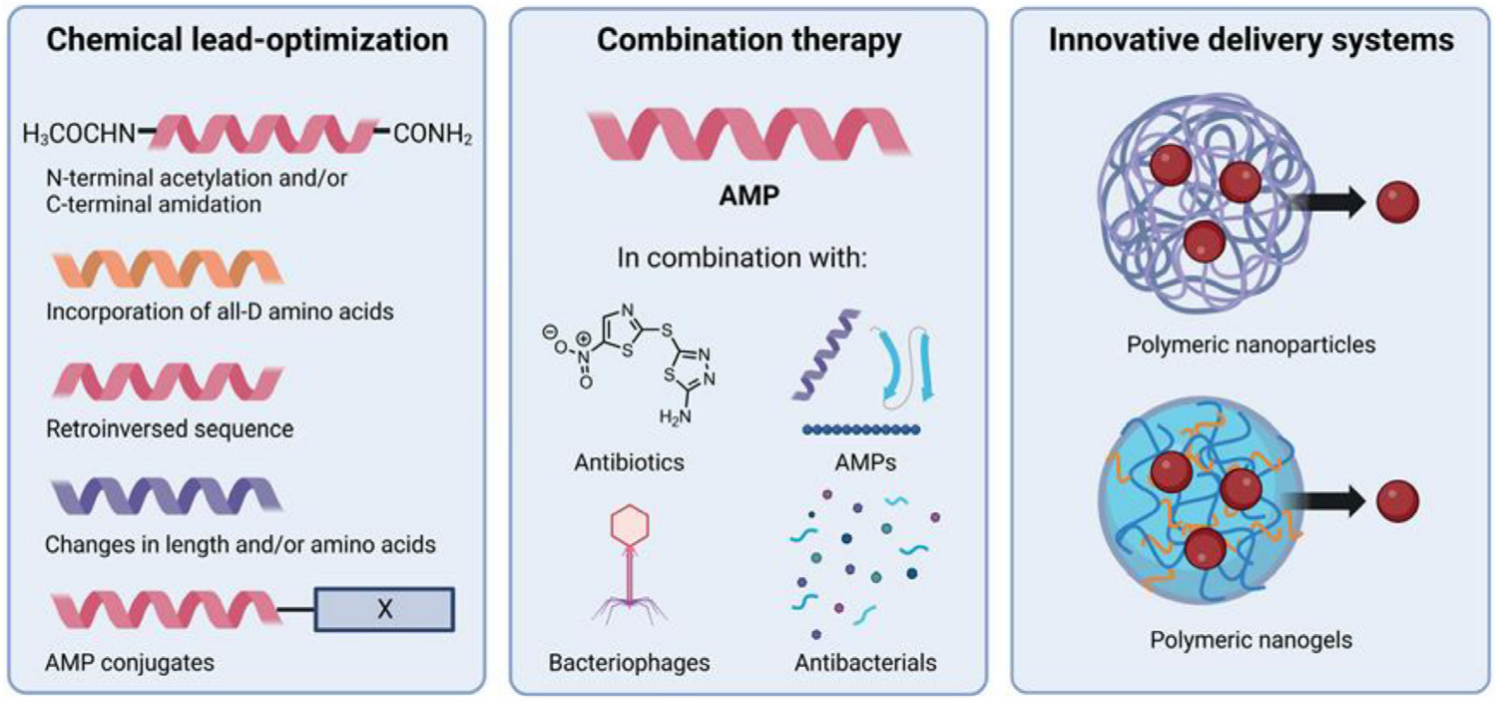
*Three strategies that can be used to further improve antimicrobial peptides (AMPs) like SAAP.* Left panel: Strategies for the chemical lead-optimization of peptides, including N- or C-terminal capping, incorporation of d-amino acids, or changes in length and/or amino acid sequence. Middle panel: AMPs can be combined with agents like antibiotics, other AMPs, bacteriophages, or other antibacterial agents that may have synergistic activities. Right panel: Innovative delivery systems that can be used to encapsulate AMPs and can be applied at the site of infection, where they release their content over time. The image was made using BioRender.

Several strategies can be considered to minimize the peptide-related limitations of SAAPs and AMPs. Such strategies include the optimization of AMP chemical leads, combination therapy, and innovative peptide delivery systems (Fig. 3).

### 5.1 Chemical lead-optimization of AMPs

Post-translational modifications of natural AMPs are common, and synthetically changing the AMP backbone is one of the many strategies that can be used to improve the pharmacokinetic and pharmacodynamic properties of synthetic AMPs. The post-translational modifications that occur in natural AMPs include N-terminal acetylation, C-terminal amidation, incorporation of d-amino acids, disulfide bridge formation, and cyclization [54]. The simplest synthetic modification is the end-capping of AMPs: N-terminal acetylation can greatly improve the proteolytic stability of AMPs (sometimes at the cost of antimicrobial activity), whereas C-terminal amidation generally improves their antimicrobial activities [55]. Moreover, the substitution of L-amino acids with D-amino acids, thereby changing the chirality, is a useful strategy to improve the proteolytic stability of AMPs [56,57]. The same holds true for retro-inversed AMPs, which have a reversed sequence and thus reversed chirality; however, their amino acid orientation in 3D space remains the same [58]. Furthermore, changes in the length and/or amino acid sequence of AMPs may improve their properties [57,59]. Alternatively, AMPs can be conjugated to other molecules to improve their pharmacokinetic and pharmacodynamic properties. The most extensively studied conjugation method is PEGylation, which involves coupling of a polyethylene glycol chain to AMPs. Generally, the PEGylation of AMPs results in decreased hemolytic and cytotoxic activities, improved proteolytic stability, reduced binding to serum proteins, and improved solubility [60,61]. Nevertheless, the reduced antimicrobial activity of PEGylated AMPs has been observed, especially upon conjugation to sufficiently long PEG chains [61,62]. The conjugation of AMPs to shorter PEG chains provides similar advantages while minimizing the loss of antimicrobial activity [63,64]. In addition, the conjugation of AMPs to cell-penetrating peptides (CPPs) or penetration enhancers has proven successful in increasing their internalization into cells and improving tissue penetration [65,66]. CPPs are a group of peptides capable of crossing cellular membranes via endocytosis and/or direct translocation [65,67]. CPPs often originate from naturally occurring amphipathic peptides with antimicrobial activity; some examples include penetratin [68], HIV-1 Tat [69], and W/R [70]. Model amphiphilic peptide is a special CPP with additional capabilities as a penetration enhancer by modulating tight junctions [71]. Notably, all the aforementioned modifications may improve the pharmacokinetic and pharmacodynamic properties of synthetic AMPs. Nevertheless, it is important to carefully review their antimicrobial activities as these modifications may undesirably decrease the antimicrobial activity of the parent peptide. The benefits and limitations of surface alteration approaches for AMPs, and how to address them, as shown in Table 1.

**Table 1:** Benefits and limitations of surface alteration approaches for AMPs, and how to address them.

AMPs	Classification	Approaches of chemical alteration	Benefits	Limitation	Way to overcome	Reference
AMP Ac-CGGP9-PEG and AMP OM19r-8	-	PEGylation	Longer half-life with lower toxicity and immunogenicity. Enhanced lifespan and biocompatibility.	Interrupted peptide structures may diminish the initial antibacterial action. Lower AMP binding capacity caused by PEG's spatial location resistance.	Design ultrashort sequences to prevent increasing molecular weight. Combine with various antibacterial drugs to enhance the efficacy of those drugs in animal studies of bacterial infection.	[72]
Caspofungin, Anidulafungin	S-Acylation N-Acylation	Lipidation	Control hydrophobicity and self-assembling tendency of AMPs. Enhances protease enzyme lifetime.	Decreased index of therapy due to cytotoxicity to normal mammalian cells.	Researchers appropriately modulate fatty acids integrated into AMP sequences to provide affordable curative medicines. In physiological settings, poor stability remains a challenge. Researchers, can overcome this limitation by introducing cleavable linkers, optimizing lipid chain length or combining lipidation with protective strategies, such as PEGylation or cyclization, which enhance stability while minimizing cytotoxicity.	[73]
Drosocin Datucin Bactenecin	C-Glycosylation S-Glycosylation N-Glycosylation O-glycosylation	Glycosylation	Enhanced AMP longevity with excellent targeting. Modified half-life expands the functional span and variety of peptides/ proteins.	AMP hydrophobicity is decreased by hydrophilic glycosylation. May decrease the initial antibacterial action.	Recombinant bacteria introduce two distinct chemical modifications into a suggested peptide, and plasmid-encoded enzymes then glycosylate them. Combining gAMP libraries with high-throughput screening studies	[54]
Daptomycin Polymyxin	-	Cyclize	Increases the hydrolytic stability of peptides Boost the ability of the target.	Expensive and prolonged production The outcomes of peptide cyclization are difficult to anticipate.	The addition of unnatural residuals to cyclic peptides. Artificial intelligence can also boost success rates by assisting with sequence and structural calculations.	[74]
6K-F17 SAAP 148	Substitution involves replacing D-amino acids and other rare amino acids. Additions (*e.g.*, acetylation at the N/C terminus)	Alteration in amino acid sequence.	AMPs exhibit enhanced stabilization and pharmacokinetic characteristics. Enhance peptide biocompatibility and bioavailability boost targeting specificity. Enhancement of antibacterial activity	Highly expensive processing. AMPs demonstrate higher epitope stiffness. Net neutralizing charge of AMP epitopes. Hydrophobic stimulation is harmful to mammalian cells and may affect the antibacterial effect of AMPs. A restricted length constrains some modifications.	Incorporating one Aib peptide at the N-terminus greatly improved plasma persistence and increased *in vivo* activities. The addition of unconventional amino acids and different chemical alterations to boost affordability and maintain structure-function interactions needed for antibacterial treatment Successful antibacterial products can be developed using database information. The improved sequences may exhibit significant sequence homology with HDPs.	[24,75]

### 5.2 Combination therapy with AMPs

The antimicrobial activity of AMPs can also be enhanced by combining them with other therapeutic agents. Drug combinations can show synergism, additive effects, or no interaction or antagonism [5,76]. Synergy between two drugs occurs when their combined effect is greater than their expected additive effect. Synergistic AMP-drug combinations are less likely to induce bacterial AMR [77,78] and thus have the advantage of reducing AMR development. Moreover, these combinations can reduce the individual drug dosage, and, in effect, reduce potential cytotoxic side effects [5,79,80]. Reasonably, favorable combinations of AMPs with first-line antibiotics would allow for more effective and shorter therapies than current antibiotic regimens. Specifically, membrane-disruptive AMPs have been successfully combined with current antibiotics, thereby facilitating antibiotic penetration into the bacterial cytosol, allowing the antibiotic to reach its target [[81], [82], [83]]. Similarly, SAAP-148 has been found to synergize with classical antibiotics such as teicoplanin [84] and demeclocycline [85]. Alternatively, AMPs have been shown to synergize with another AMP, and the addition of a third AMP to the combination further improves these synergistic activities [79]. Moreover, a recent study on bacteriophages showed that they can synergize with AMPs [86]. Finally, other antibacterial agents could also synergize with AMPs, like phage endolysins [87], histones [88] or silver nanoparticles [89]. In the context of bacterial biofilms, AMPs can be combined with agents that inhibit biofilm formation (*e.g.*, inhibitors of quorum sensing [90]) or degrade and/or disaggregate the biofilm matrix (*e.g.*, matrix-degrading enzymes [91] and chelating agents [[92], [93], [94]]). Finally, combinations of conjugates of AMPs and the above-mentioned antimicrobials could also be considered as they may have several advantages in the combined application of the two agents [95].

### 5.3 Innovative delivery systems for AMPs

Another strategy to improve the therapeutic potential of AMPs is their encapsulation in drug delivery systems (DDS) [7]. Since the introduction of the first controlled-release drug formulation in the 1950s [96], drug delivery technology has advanced significantly, with a wide range of DDS now available for AMP encapsulation. DDS can be categorized as either inorganic materials (*e.g.*, metal-based nanoparticles and mesoporous silica nanoparticles) [97] and organic materials (*e.g.*, lipid-based nanoparticles, polymeric nanoparticles, and polymeric nanogels) [98].

Synthetic or natural organic substances, including polymers, proteins, and lipids, are often used to form organic nanocarriers with dimensions ranging from 1 to 1000 nm. They are biocompatible, biodegradable, and can accommodate various drug molecules [99]. Natural substances and nanocarriers containing AMPs have been classified into two types: those with incorporated AMPs and those with combined AMPs [100]. The first confines AMPs in a carrier matrix, whereas the latter incorporates AMPs into the structure of organic molecules. Researchers frequently use organic nanocarriers, such as nucleic acids, lipids, and polymers, to regulate AMP delivery, enhance drug distribution, and reduce cytotoxicity. frequently use organic nanocarriers, such as nucleic acids, lipids, and polymers, to regulate AMP delivery, enhance drug distribution, and reduce cytotoxicity [99,100].

Similarly, inorganic nanocarriers possess specific properties that make them ideal candidates for diagnostic and therapeutic applications [101,102]. Their surface features can be modified through shell development and ligand exchange, including targeting ligands, PEG coatings, and the insertion of responsive moieties, thereby improving biodistribution, prolonging circulation half-life, and increasing cellular uptake [101,102]. Inorganic nanocarriers containing AMPs can be classified as nanotubes or metallic nanoparticles. Nano-drug delivery platforms stabilize AMPs through non-covalent interactions, including hydrophobic contacts and ionic-complementary interactions, thereby enhancing their pharmacokinetic properties and extending their *in vivo* efficacy [101,102]. Nanocarriers, such as metallic nanoparticles (*e.g.*, gold, silver, and zinc oxide), mesoporous silica nanoparticles, and layered double hydroxides, may provide structural support and protect AMPs from enzyme degradation, thereby extending their bioactivity under physiological conditions [5,7,101]. The advantages and limitations of various nanocarrier delivery strategies of AMPs for biofilm inhibition and ways to overcome them are shown in Table 2.

**Table 2:** Advantages and limitations of various nanocarrier delivery strategies for AMPs to inhibit biofilm formation.

Nanoparticle type	Organization	Features	Advantages	Limitation	Way to overcome	Reference
Solid Lipid nanoparticles	Lipid-based nanoparticles that dissolve at temperatures above 40 °C.	A solid lipid matrix with solid lipid nanoparticles. Solid lipids submerged in oil droplets that have been stabilized by suitable surfactants make up nanostructured lipid carriers and can carry hydrophilic or hydrophobic substances.	Bio degradable, and non-toxic NPs capable of carrying a wide range of biological molecules, and can be surface-modified for precise delivery. Their colloidal size and slow-release properties make them excellent for oral, ophthalmic, and parenteral delivery, with topical treatment proving especially effective.	The lipophilic nature of these two nanoplatforms limits their ability to retain polar peptides, a common problem.	Nanostructured lipid carriers or surface changes may enhance the persistence of polar peptides in SL nanoparticles.	[104,115]
Liposomes	Spherical nanostructures made of phospholipids and cholesterol forming a lipid bilayer surrounding a water-based core.	Liposomal particles vary in dimensions between 0.025 and 2.5 μm and are characterized by a variety of bilayers (lamellae) around a liquid core.	Liposomes have many benefits, notably amphiphilicity, excellent solubility, strong dispersion stability, drug protection against environmental influences, biocompatibility, biodegradability, and the potential to deliver compounds intracellularly. Furthermore, its phospholipid composition confers a strong affinity for biological membranes, thereby significantly increasing cellular drug absorption.	Lipid bilayers are susceptible to chemical deterioration, oxidation, and hydrolysis, which may compromise their systemic durability and integrity. Liposomes are also subject to physical instability, such as accumulation, merging, or collapse, which may affect vesicle diameter and cause drug leakage. Their other disadvantages include possible allergic reactions, high production costs, and lengthy manufacturing periods.	The use of stabilizers, polymer coatings, cryoprotectants, and optimized production procedures can overcome liposome constraints, enhance longevity, and lower costs.	[116,117]
Dendrimers	Made of three fundamental portions: a base, monomeric strands, and terminal functional units. These branches are connected to the base in a globular form, while the terminal units are outside ligands that bind to them.	Three-dimensional, hyperbranched polymeric systems that are usually between 2 and 5 nm in size.	Researchers may make terminal clusters with target-specific components to increase the number of particles at the intended site of activity. Its globular form enables drug encapsulation by creating nanodomains with distinct characteristics at the core and the periphery.	Tumor cells absorb minimal nanoparticles, and the immune response clears them quickly.	Surface activation, targeted ligands, and stealth surfaces to improve tumor absorption and decrease immune clearance	[118,119]
Polymeric nanoparticles	Polymers in the shape of nanocapsules or nanospheres.	Size ranges from 1 to 1000 nm.	Enables the regulated distribution of different types of medications based on variations in a scaffold formulation. The surface formulation of polymeric particles (which may be enhanced by ligands) enables drug delivery to a specific region, reducing toxicity and boosting therapeutic effectiveness. Additionally, they could be delivered in several ways.	Most processes involving premade polymers use chemical solvents, which can be poisonous and environmentally hazardous, requiring solvent cleanup.	To ensure safety and a sustainable environment, consider using non-solvent processes, eco-friendly fabrication strategies, and biodegradable materials.	[120,121]
Inorganic/ metallic nanoparticles	Metal oxides	They can consist solely of metal oxides, metals, or metal salts and vary in size from 10 to 100 nm.	Being positively charged, metals may interact strongly with bacterial membranes, resulting in cell damage, cytoplasmic permeability, and entry of metal ions into bacteria, which may trigger metabolic challenges. Such tiny particles inhibit biofilm formation through entering bacterial cell membranes, affecting quorum sensing, and disrupting interactions with the extracellular polymeric matrix.	The toxic effects of the substances used in nanoparticle fabrication, along with their poor biodegradability, limit their application.	Eco-friendly coatings, sustainable fabrication processes, and biodegradable composites can help minimize cytotoxicity and increase safety.	[104,115]

Several limitations of AMPs can be circumvented by nano-scaled DDS through i) protection of AMPs from proteolytic enzymes and prevention of binding to plasma and/or serum proteins, thus improving the stability and bioavailability of AMPs [103,104]; ii) sustained release of AMPs, which reduces the cytotoxicity associated with DDS [105,106]; iii) assisted transport of AMPs across cellular membranes, thus improving intracellular uptake [106,107]; and iv) improved biofilm penetration and intracellular retention [17,[106], [107], [108]]. The physicochemical properties of these nanoscale DDS (*e.g.*, material composition, particle size, and surface charge) affect their pharmacokinetic profiles and cellular interaction [109]. Thus, depending on the application, one delivery system may be preferred over another.

For instance, nanogels have received great interest as versatile DDS for cutaneous application in skin wounds because of their unique properties resulting from the combined features of nanoparticles and hydrogels. Their soft nanoscale particles are formed when water-soluble polymers (natural or synthetic) are cross-linked in 3D space, while the resulting nanogels can absorb large amounts of water or biological fluids into the formed network while maintaining their structure [110]. The large number of hydrophilic groups in the polymeric backbone of nanogels allows highly efficient AMP encapsulation and provides high biocompatibility [111]. Furthermore, nanogels are considered ideal DDS for skin wound infections because of the following properties: i) their high water content prevents wound dehydration and creates a moist environment beneficial for wound healing [112,113]; ii) their soft texture and non-adhesive properties allow for patient-friendly application and removal without interfering with the wound bed; and iii) their porous three-dimensional structure allows for the exchange of oxygen, which is highly important for numerous metabolic processes involved in wound healing [114]. Preclinical *in vitro* and *in vivo* investigations have demonstrated the curative properties of various organic and inorganic nanocarrier-loaded AMPs, as shown in Table 3.

**Table 3:** Features and significant outcomes from various *in vitro* and *in vivo* investigations employing AMP-loaded inorganic and organic nanocarrier platforms.

**AMPs**	Nanocarrier	Size	Targeted pathogens	Dosage	Major results	Reference
LL-7	Gold nanocarriers	73.7 ± 12.6 nm	Gram-negative pathogens	15 μg/mL (*in vitro*) 240 μg (*in vivo*)	Improved antibacterial action, accelerated angiogenesis, and prevented bacterial contamination in diabetic ulcers, rapid wound healing times, accelerated re-epithelialization, enhanced granulation tissue development, and elevated VEGF expression.	[122]
Polymyxin B	B-polysaccharide-nanoparticles	154.2 ± 1.4 nm	*Pseudomonas aeruginosa*	MIC (2 μg/mL) and MBC (4–8 μg/mL)	Greater safety and efficacy.	[123]
Bacitracin	Polymeric hydrogel		MRSA	MIC (3.125 μg/mL) and MBC (12.5 μg/mL)	Improved safety and enhanced anti-inflammatory, anti-biofilm, and accelerated wound healing.	[7]
Dpep	Silver nanocarriers	1.25 nm	*S. aureus*	13 μM	Association with the bacterial membrane targets LPS, which has a 100-fold greater inhibitory effect.	[124]
β-cyclodextrin	Mesoporous Si nanoparticles	90–110 nm	*P. aeruginosa*	50 mg/L (*in vitro*) and 250 mg/kg	Enhancing antibacterial activity while reducing target tissue loss and inflammatory responses.	[125]
Polymyxin B	Lipid nanoparticles	98.64 ± 0.914 nm	*A. baumannii*	1 μM (*in vitro*) and 10 μM (*in vivo*)	It damaged microbial cell walls, thereby increasing ROS levels, and controlled wound recovery through granulation tissue development, collagen remodeling, and promotion of angiogenic growth and M1/M2 macrophage polarization.	[126]
Polymyxin B	Niosomes	170.2 ± 2.3	*K. pneumoniae*	64 µg/mL (*In vitro*) and 2.0–2.5 mg/kg (*in vivo*)	Inhibited bacterial envelopes by addressing LPS, allowing combinatorial death using polymyxin B, and observed no harm *in vitro* and *in vivo*.	[127]
Esculentin	PLGA nanoparticles	282 ± 4	*P. aeruginosa*	0.1 mg/kg (*in vivo*)	Stronger antibacterial effects, resulting in a 3-log reduction in lung bacterial growth for up to 36 h.	[128]
Colistin	Chitosan-lipid nanocarriers	485.0 ± 9.8 nm	*P. aeruginosa*	16 µg/mL (*in vitro*)	Increased antibacterial activity (4-fold) in pathogenic and antibiotic-resistant strains.	[129]
Nisin	Ag Nanoparticles	588 ± 191 nm	*P. aeruginosa, K. pneumoniae, E. coli*, and *Salmonella typhimurium*	-	AgNanoparticles containing nisin suppressed Gram-negative bacteria. Thus, a wound dressing with antibacterial effect might be fabricated.	[130,131]

#### 5.3.1 Stimulus-responsive systems

Scientists have designed stimulus-responsive or intelligent curative platforms that can alter the physical and chemical properties of therapeutics in response to specific environmental or externally imposed stimuli. Targeted therapies can therefore eradicate biofilms as scientists can spatially and temporally regulate the administration of antibiotics or the application of physical forces to minimize systemic toxicity and prevent the development of bacterial resistance [132]. In biofilm-related diseases, such strategies allow rapid and targeted therapy at the affected site, circumventing the inefficiencies and constraints of traditional coatings that persistently and non-specifically deliver drugs. The interplay of nanomaterials, AI, stimulus-responsive systems, and enhanced antibiofilm strategies enables rapid targeted therapy at the affected site [133,134].

Internal stimuli leverage microenvironmental factors unique to biofilms, such as regional pH, enzyme function, and metabolite-driven activity. Biofilms frequently have an acidic environment (pH 5.05 to 6.5) due to anaerobic bacterial metabolism, which permits pH-responsive vehicles to form or disintegrate biofilms [135]. Enzyme-responsive platforms have been shown to selectively transport cargo over *S. aureus* biofilms [136]. Furthermore, metabolite-sensitive systems can detect quorum-sensing or reactive oxygen species during biofilm development, resulting in targeted therapies [137].

Conversely, external factors employ non-invasive stimuli to engage in curative functions. Biofilm elimination using light activation techniques, including photodynamic therapy and photothermal therapy, has shown considerable potential. In this regard, the introduction of near-infrared light-activated nanostructures could aid in maintaining targeted hyperthermia or the production of reactive oxygen species, potentially disrupting biofilm formation [138]. Furthermore, magnetic field-activated approaches enable the electronic removal of drug distribution or direct heating via magnetic hyperthermia [17]. In addition to temperature-responsive vehicles, poly(N-isopropylacrylamide) shrinks and extends because of the reversible phase transitions induced by moderate hyperthermia, allowing efficient drug delivery and mechanical breakage [139].

## 6 Critical limitations in advancing AMP-derived nanoformulations into healthcare settings

Because AMP-derived DDS offer various benefits over their free-form AMPs, without DDS/without chemical modification, researchers must overcome significant hurdles in developing sustainable therapeutic materials for treating infectious diseases. One of their disadvantages is the lack of standard tests in bio-appropriate situations, which enables better assessment and comparison among DDS. Moreover, preparing and evaluating shelf-stable macroformulations are crucial for the fabrication of efficacious healthcare treatments. The following sections discuss these issues in detail.

### 6.1 Lack of standardized tests

Despite numerous studies on the fabrication and *in vitro* characterization of DDS, the biological effects of AMP-loaded preparations have not been extensively assessed. One of the most frequent drawbacks is the inappropriate use of controls. In some instances, peptide-loaded platforms are compared with non-loaded platforms without therapy rather than peptide treatment. These comparisons fail to reveal whether peptide intrinsic antimicrobial activity decreases during encapsulation. Reliable *in vitro* and *in vivo* assessments are essential to establish the antibacterial efficacy and safety of the delivery method used. Researchers have primarily examined antibacterial agents using broth microdilution minimum inhibitory concentration (MIC) analysis, or zone inhibition assays [140]. In particular, these two strategies rely on planktonic bacteria, whereas bacterial growth include neither planktonic nor biofilm-linked bacteria nor persisters that tend to be resistant to therapy. Several complicated *in vitro* procedures are currently in use, including killing tests for planktonic bacteria and premature and mature biofilms. Employing an applicable ecosystem that simulates a clinical scenario to determine the feasibility of a delivery mechanism for specific uses may improve the outcome of these studies. This ecosystem includes host cells, such as 3D elastic collagen scaffold systems, immune cells, and biological fluids, such as proteolytic enzymes, urine, or plasma proteins. Several studies have shown that these materials regulate the antibacterial effect of AMPs *in vivo* [41,53,141,142]. Similarly, researchers must assess cytotoxicity in more complicated *in vitro* cell models that resemble infectious scenarios, including 3D skin designs [143]. Furthermore, complex yet useful *ex vivo* external wound systems are currently being developed, such as incision wound studies [144], burn wound studies [52], tape-stripped skin, and intact skin studies [143,145], all of which use human skin. Such studies offer performance evaluations that are free of the ethical issues that arise during *in vivo* trials.

Furthermore, when suitable *in vitro* and *ex vivo* evaluations are completed, *in vivo* trials are required to assess the pharmacokinetics and pharmacodynamics of DDS and immune reactions, in addition to antibacterial efficacy assays and safety determination. Before selecting an animal model, it is essential to thoroughly analyze the alternatives available. For instance, topical wound studies on mouse and pig skin have been performed [146,147]. However, if the purpose is to cure biofilm formation in lung tissue in the case of cystic fibrosis, finding appropriate models could prove challenging. Scientists have genetically manipulated mice to exhibit cystic fibrosis-like symptoms; however, these models failed to adequately represent the severity of human infection, prompting scientists to build the concept in larger species such as guinea pigs and ferrets [148]. Furthermore, the inadequacy of standards for administering nanoparticulate complexes *in vivo* remains problematic, as there is a standard description of nanoparticle dosage in biological specimens (*e.g.*, blood, urine, and organs) [149]. Researchers have reported achievements with lipid and polymeric AMP delivery systems *in vivo*; however, additional research is required to comprehensively understand their *in vivo* actions in an era when AMR is becoming more prevalent. Thus, moving quickly from *in vitro* and *ex vivo* studies to *in vivo* assessments is critical.

### 6.2 Limitations of long-lasting formulations and their analyses

Although nanoparticle-derived methods and coatings have demonstrated excellent efficacy both *in vitro* and *in vivo*, the fabrication of long-lasting liquid, gel, or solid dosage forms is still required for DDS applications in healthcare settings. The storage durability of AMP-derived therapeutics is critical because of the reactive character of AMPs, which are susceptible to breakdown and hydrolysis, as well as the interactions between AMPs and macroformulations. For example, incorporating SAAP-148 into a hypromellose gel carrier has been shown to diminish the effectiveness of the peptide: increased viscosity decreased its activity [52]. Dijksteel et al. also investigated several readily accessible wound dressings, mainly traditional gauze, drenched in the peptide SAAP-148 and discovered that the wound dressing composition significantly inhibited the action of the peptide. This was most likely due to AMP binding, which lowered the amount of peptide accessible for bacterial interactions.

Nonetheless, some macroformulations yielded positive outcomes. In drug-resistant *A. baumannii*-associated burn wounds, hydrogels produced by cross-linking the AMP epsilon-poly-L-lysine moiety with catechol dramatically decreased bacterial prevalence by >4 log [150]. Chitosan- and polycarbonate-based hydrogels with AMPs also demonstrated promise as wound dressings [[151], [152], [153]]. Furthermore, hydrogel wound dressings have demonstrated the ability to aid wound clearance by rehydrating inactive tissues [7], which is required for wound recovery, rendering them an ideal macroformulation for wound dressing production. However, despite researchers finding positive outputs for peptide solutions in some macroformulations, no one has reported findings specifically regarding DDS macroformulations. These results highlight the importance of evaluating the final dose of the AMP delivery system, preferably *in vivo*, as it can significantly affect efficacy.

## 7 Therapeutic uses of AMP delivery systems

**Fig. 4:**
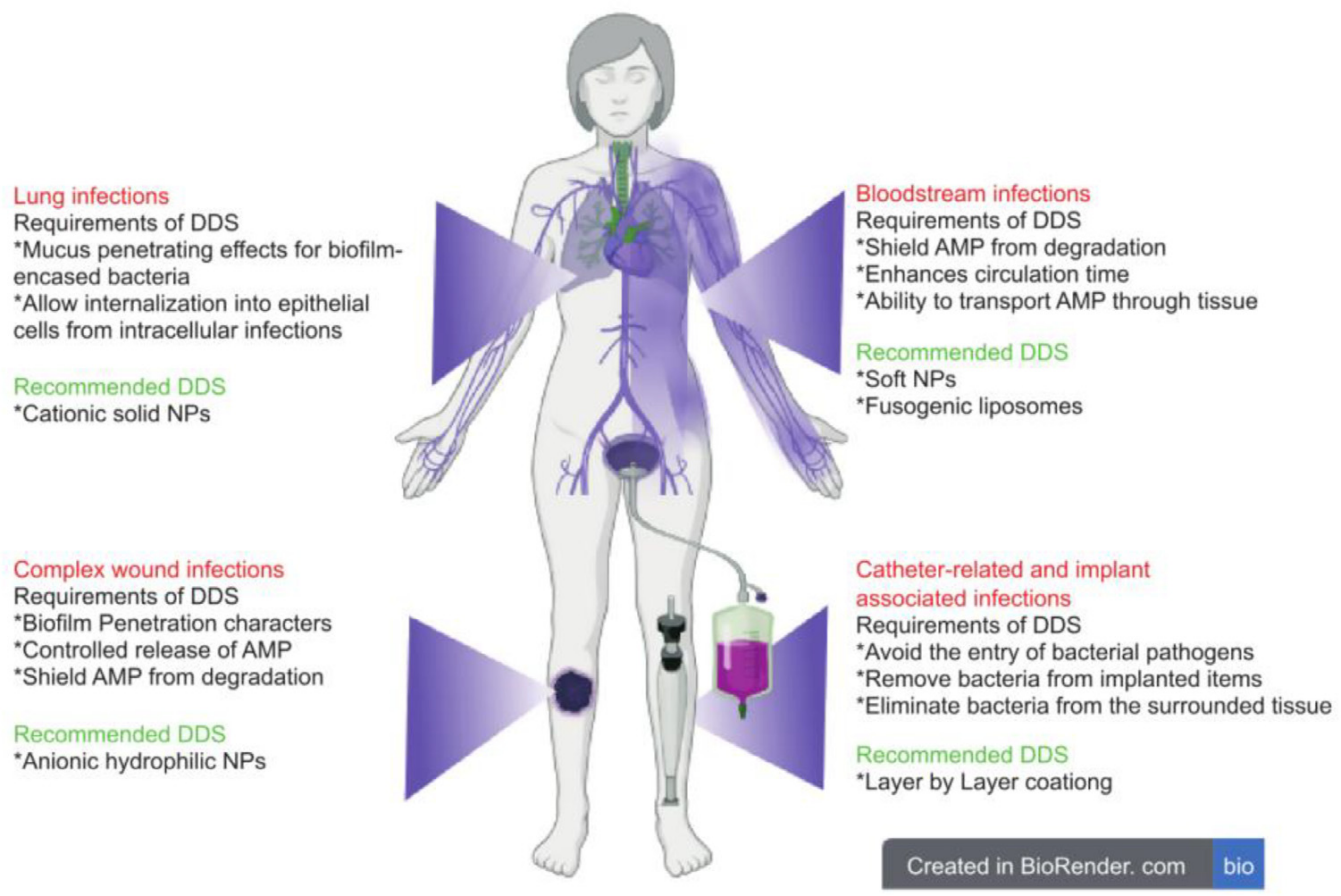
*Summary of the required and preferred drug delivery systems for different diseases.* Figure is based on van Gent et al. [202]. The image was made using BioRender.

Another significant challenge is the selection of AMP-mediated DDS for the treatment of various illnesses. In this section, we aim to establish whether the polymeric/lipid AMP administration method or coating has been successfully used to treat the most serious and difficult-to-cure illnesses. Fig. 4 summarizes the required and preferred DDS for various diseases.

### 7.1 Systemic and persistent diseases

For the treatment of bloodstream-circulating bacteria and deeper organ diseases, a DDS that shields the peptide against enzyme breakdown and quick elimination from the bloodstream, while also being systemically administered, is ideal. Smooth nanoparticles, especially nanogels, could be helpful for this application because of their properties, including deformation, improvement in blood flow, and facilitation of their movement across tissues to the infected area [154]. Fusogenic liposomes are especially useful because they can bind to the outer membrane of bacteria and directly provide large quantities of AMPs to the bacterium [155]. However, liposomes can rapidly enter the bloodstream. PEG wrapping on liposomes, which render the exterior of the resulting molecule hydrophilic, was discovered to enhance their blood persistence and specificity in diseased lungs [156]. Moreover, PEG-encapsulated PLGA nanoparticles were demonstrated to promote blood flow [157]. Another study reported that the diameter of systemically delivered nanoparticles should be greater than 20 nm to prolong blood circulation time and prevent kidney filtration, and less than 100 nm to prevent filtration by the spleen and liver [158].

### 7.2 Diseases associated with catheters and implants

Treatments involving catheter or implant use can spread infectious diseases. Therefore, there is considerable demand for AMP-releasing coatings to prevent or alleviate biomaterial-related illnesses. Coating implant items with materials containing AMPs may prevent infection and reduce post-surgical problems, whereas releasing the peptide may kill bacteria that currently exist in tissues close to an implant. Although excellent outcomes have been recorded for various coatings *in vitro*, layer-by-layer coating proved to be the most efficient for chronic disease mitigation, with controlled release being recorded for as long as four months for specific layer-by-layer coatings [159].

### 7.3 Pulmonary and intracellular infections

In certain respiratory diseases, including chronic bronchitis and cystic fibrosis, an external delivery approach via inhalation may be superior for locally releasing significant amounts of AMPs. After inhalation, AMPs must pass across the pulmonary mucus and bacterial biofilm to reach the bacteria. Hence, mucus-penetrating nanoparticle technology may be helpful because mucociliary elimination continuously removes pulmonary mucus. This mechanism may enable permeation across the mucus layer, delivering large amounts of AMPs to the infected site. Furthermore, if the goal is to cure an intracellular disease, including *Mycobacterium tuberculosis* infection, a route of administration that involves absorption by epithelial cells is preferred. Smaller nanoparticles (<20 nm) stimulate absorption but not endocytic processes [160,161], whereas nanoparticles larger than 100 nm exhibit higher cytotoxicity or metabolic burden *in vitro* and *in vivo* [162]. Even larger particles (approximately 200 nm) are often designed for intracellular distribution. Furthermore, cationic and solid particles appear to be more effective, whereas negatively charged and softer particles exhibit much lower cellular uptake across various cells. Identical findings were obtained with PLGA nanoparticles, where altering their outer charge from negative to positive dramatically increased their cytoplasmic transport [163,164]. The use of chitosan, a cationic polymer, to coat particles such as liposomes or PLGA nanoparticles also considerably improved their intracellular transport [165,166]. Substantial research on nanogels has demonstrated high cellular absorption and antibacterial efficacy against intracellular infections, considering their generally softer properties.

### 7.4 Complex wound infections

Complex wound diseases, including burn wound complications, fracture-associated illnesses, and prosthetic arthritis, are linked to biofilms that shield bacteria from the host immune system, significantly improving AMR [7]. It may be necessary to address such injuries surgically by removing the most damaged tissue and administering strong antimicrobial therapies. Standard prophylaxis employing cationic antibacterial agents such as gentamicin or AMPs has proven ineffective because of matrix adherence [167]. A DDS that can be applied topically and can penetrate the biofilm, releasing AMPs to bacteria in biofilms for a longer time, would be ideal. Negatively charged and hydrophilic particles [80,168], including colistin-infused nanostructured lipid carrier [169], have been shown to penetrate and accumulate in bacterial biofilms. Furthermore, prolonged release is preferable in this context because it lowers the number of wound dressing replacements needed and the accompanying discomfort. As stated previously, the acceptability of a DDS for specific uses is determined solely by reporting a particular delivery method. Contrasting opinions on the qualities needed for specific uses highlight the importance of comparing multiple DDS to establish their most desirable features for such purposes. Hence, consecutive evaluations of DDS under consistent settings using similar concepts are required. This knowledge allows the selection of the most appropriate DDS for specific circumstances, significantly increasing the chances of successfully developing a product for clinical use.

## 8 Future perspectives

Although substantial advances have been made in AMP delivery methods, further research is required to develop nanoparticle-based solutions or coatings for clinical use. A notable limitation of several previously described DDS is their inferior encapsulation efficacy. This could be related to the increased burden due to the required purification procedures and high cost due to considerable peptide loss during purification. Encapsulation efficacy may be enhanced by adjusting the formulation characteristics to fit the encapsulated drug or molecule, as demonstrated using liposomes [170], PLGA nanoparticles [171], and nanogels [111]. Additionally, the functional characteristics of the system, including the AMP release rate from the nanoparticles, can be adjusted by varying the formulation method of the delivery vehicle, including the amount of lipids or the molecular mass of the polymer used. The latest progress in the development of AMP delivery methods appear to be the creation and growth of hybrid AMP delivery techniques, in which the outermost layer of the particle is modified with PEG, biofilm-penetrating ligands, or CPPs to enhance delivery and intracellular absorption while overcoming any potential systemic drawbacks. Enhancing prior delivery methods efficiently is an ideal solution for boosting AMPs targeting the intended action area, while leveraging the existing understanding of current delivery systems. Furthermore, established standards and long-term implementation of these technologies are also required. Consequently, there is a pressing need to develop *in vitro* and *ex vivo* testing techniques along with more appropriate *in vivo* disease models, ideally assessing long-lasting forms of these drugs to evaluate the efficacy and safety of such compositions.

### 8.1 AMP-loaded nanocarriers: current clinical trial progress

Despite substantial preclinical research, no human clinical studies using nanocarriers containing AMPs targeting bacterial infections have been reported. Other AMPs without nanocarriers, such as dusquetide (IMX942, SGX942) [47,[172], [173], [174]], melimine and Mel4, p2TA (AB103, reltecimod) [175], and PL-5 (peceleganan) [172,173], have undergone various stages of human validation, but these did not employ nanocarriers. Conversely, nanocarrier-based antibiotics have the potential to regulate AMR pathogens [[176], [177], [178]]. Hence, although many preclinical and clinical trials have provided significant translational precedents, AMPs loaded nanocarriers are still in the preclinical stage as unique therapeutic alternatives.

In preclinical testing, many AMP-loaded nanocarriers have demonstrated potential as drug delivery strategies for Gram-negative infections and have shown first-in-human efficacy. LL-37-loaded chitosan-capped liposomes demonstrated improved antibacterial activity and persistence in *E. coli* and wound infection studies, indicating their significant promise for topical use [179,180]. Self-assembling nonapeptides, especially N3FT and F3FT, show both antibacterial and anti-inflammatory effects by disrupting bacterial membranes, generating reactive oxygen species, and eradicating intracellular bacteria, demonstrating improved biocompatibility and providing an innovative and effective approach against pathogenic bacteria [181].

### 8.2 AI-assisted antimicrobial peptide design

Historically, AMP development has depended on iterative laboratory workflows, chemical modifications, diverse alteration strategies, phage displays, and solid-phase synthesis. Although these experimental approaches are indispensable for advancing initial hits to optimized leads, they are frequently cost-prohibitive because they require large-scale production and extensive manual screening [182]. For instance, creating point mutations in the primary sequence of a peptide is technically straightforward, yet the subsequent optimization pipeline is expensive [47]. High-throughput platforms, such as phage display and solid-phase peptide synthesis, can produce vast peptide libraries and yield valuable design information, but they are time-intensive and costly [183]. Specifically, both the chemical synthesis of candidate AMPs and antimicrobial activity assays required for their optimization consume substantial resources and time. Consequently, conventional methods face significant challenges in terms of the efficiency and cost of AMP discovery and design.

The rapid evolution of computing has significantly enhanced artificial intelligence (AI) and machine learning (ML) tools for AMP identification and design, thereby improving throughput and lowering costs. ML, a core component of AI, trains models on diverse datasets to rapidly and accurately identify bioactive peptide sequences from numerous candidates [184]. For example, Table 4 lists several ML frameworks used for AMP prediction and classification, such as AntiBP2, iAMPCN, and AMPDeep, which employ various algorithms, including XGBoost, random forests, convolutional neural networks, support vector machines, long short-term memory networks, and transformer architectures. These models are trained on repositories such as APD, DBAASP, DRAMP, and YADAMP, and applied to tasks ranging from short-peptide identification and membrane-specific activity prediction to AMP classification and hemolytic activity forecasting.

**Table 4:** Databases and machine learning algorithms for antimicrobial peptides.

Algorithms	Training data sets	Names of Designs	Uses	Reference
Long short-term memory (LSTM)	LAMP, APD3, PlantPepDB, BaAMPs, Bio-PepDB, CAMP, DBAASP, DRAMP	AMP-EBiLSTM	Predicting antimicrobial peptides accurately	[188]
Transformer	DBAASP, Swiss Prot, Hemolytik	AMPDeep	Forecasting the hemolytic action of AMPs	[189]
Convolutional neural network (CNN)	CAMP, APD3, dbAMP, DRAMP, etc.	iAMPCN	Finding AMPs and comparing how they work relative to existing prediction techniques	[190]
Support Vector Machine (SVM)	APD	AntiBP2	AMP prediction and classification	[191]
Lasso and Decision Tree, Random forests (RF), SVM	PG-1 Library (more than 5.7 million PG-1 variations)	dmSLAY	Accurately predict membrane-specific actions for more than 5.7 million protegrin-1 variants	[186]
LSTM, CNN, RF, XGBoost	YADAMP, APD, DADP, DBAASP, and DRAMP	SMEP	Finding a short but efficacious peptide	[185]

The application of advanced ML models has accelerated AMP discovery by enabling the rapid *in silico* screening and prediction of antimicrobial properties. Huang et al. used a machine-learning pipeline to screen a virtual peptide library of 6–9 amino acids and identified three AMPs—CRRI3, CRRI4, and CRRI7—that exhibited potent activity against multidrug-resistant bacteria, including MRSA, with MICs of 8–32 μg/mL; these peptides also showed efficacy *in vivo*, indicating therapeutic potential [185]. Structure-aware ML strategies further reveal the relationships among the sequences, structures, and functions of AMPs. Researchers have built highly accurate models for predicting specific AMP categories by analyzing features such as dipeptide frequencies and physicochemical descriptors. Randall et al. predicted membrane-specific roles for over 5.7 million protegrin-1 variants using ML and identified a modification that markedly reduced toxicity while retaining efficacy in a mouse intraperitoneal infection model [186]. Beyond activity and structural prediction, AI can also assess therapeutic attributes, antimicrobial potency, ADMET properties, resistance potential, and can even generate novel AMP candidates through generative modeling [187]. Thus, transitioning from traditional wet lab-centric workflows to AI-assisted designs can overcome the many limitations of classical approaches and cost-effectively accelerate antibiotic discovery amid rising AMR.

Despite these advantages, AI performance depends heavily on the availability and quality of experimental data. Moreover, the limited volume of high-throughput AMP datasets constrains model training and reduces predictive accuracy. Models trained on specific datasets may generalize poorly to novel sequence spaces, posing a significant hurdle in navigating the enormous combinatorial landscape of peptides. The inconsistent adoption of open science practices in AI-driven antibiotics research further undermines its transparency and reproducibility. To accelerate preclinical progress, the field must develop open and reproducible workflows and standardized benchmarks.

Bridging computational predictions with experimental validation is essential. *In silico* candidates must undergo rigorous synthetic validation, activity assays, toxicity testing, and animal studies to confirm their therapeutic potential. Closer collaboration between computational scientists and experimentalists shortens the design test cycle and reduces the effort wasted on false positives. Incorporating techniques such as transfer learning, active learning, and generative modeling can also help explore underrepresented regions of the sequence space more efficiently, lowering the reliance on exhaustive wet lab-based screening. Explainable AI methods are also critical because interpretable models reveal which sequence motifs or physicochemical properties drive activity or toxicity, guiding rational design and increasing confidence in model outputs.

In summary, although traditional laboratory methods remain foundational for AMP development, AI and ML offer transformative potential to accelerate their discovery, reduce costs, and enable more targeted approaches. Realizing this promise requires investments in expensive, high-quality AMP databases, wider adoption of open science standards, improved model interpretability, and tighter integration of computational predictions with empirical validation steps, which can accelerate the conversion of virtual peptide designs into effective, clinically relevant antimicrobials.

### 8.3 Current gaps and research prospects

AMP-loaded nanocarriers may provide a flexible and highly effective replacement for AMPs and standard antibiotics. However, many societal obstacles persist, hampering their therapeutic progress. Despite the rapid expansion in preclinical AMP research, considerable translational gaps remain. Most studies have focused on demonstrating efficacy across a range of *in vitro* and *in vivo* settings; however, no human studies of AMP-loaded nanocarriers have been conducted to date [192]. This gap highlights significant and outstanding difficulties, such as non-selectivity, proteolysis tolerance, limited therapeutic horizons, and future safety challenges that impede regulatory progress [[193], [194], [195]].

Problems associated with the immune responses to AMP-loaded nanocarriers also remain poorly understood. AMPs and nanocarriers, such as polymers, lipids, or metal-based frameworks, can stimulate innate as well as adaptive immune responses, thereby facilitating efficient immunological elimination, particularly at higher doses [[193], [194], [195], [196]]. Some strategies, including the detection, improvement, and successive personalization of AMPs employing various sophisticated ML techniques and PEGylation, are also being investigated. However, no common or systematic immune profiling technique exists for these strategies [194,195,197]. Overall, these difficulties underscore why AMP-loaded nanocarriers remain in the preclinical phase, and there remains a critical need for novel therapeutics to combat multidrug-resistant illnesses [196,197].

To overcome these translational challenges, future trials should focus on standardized assay frameworks, immunologically guided designs, and scalable production methodologies [194,195,198]. The combination of computational ML and deep generative AI-mediated algorithms with peptide engineering can accelerate AMP detection while maintaining optimal biochemical and carrier compatibility [197]. Microenvironment-responsive stimuli, including ROS, pH, and enzymes, must be used to selectively target AMP function at infection points while minimizing systemic contact [198]. Moreover, the use of innovative stealth or activated nanocarrier approaches reduces long-lasting immunogenicity and cytokine profiles and allows initial preclinical assessments of complement activation, immunological memory, and drug antibodies [198].

Finally, *in vitro* and *in vivo* performance testing must be standardized using specific benchmarks for particle charge, size, and drug release characteristics. Adopting modern microfluidic systems and production methods would also reduce costs, enable greater scalability and repeatability, and reduce the gap between wet lab advances and clinical results [198,199]. Initial-stage clinical studies on bacterial infections should also prioritize targeted delivery using biomarker targets and bacterial load assessment, and include safety precautions to mitigate systemic impacts [198,199]. Despite their limitations, AMP-loaded nanocarriers can pave the way for first-ever human trials targeting AMR bacteria by addressing production feasibility and reducing off-target immune responses [200].

## 9 Conclusions

The increasing emergence of bacterial resistance to standard antibiotics has shifted the attention of researchers towards AMPs. Nevertheless, inadequate physicochemical features, poor efficacy, toxicity information, and high costs have hampered the development of several AMPs as therapeutic medicines. Nanoparticle-based AMP treatments and coatings have demonstrated encouraging effects *in vitro*, facilitating the delivery of AMPs not only through bacterial biofilms and in proximity to bacteria but also into cells to treat intracellular infections. Immediate priorities include standardized *in vitro* and *ex vivo* testing parameters, relevant *in vivo* infection models, and the development of shelf-stable formulations to assess their long-term safety and performance. Coordinated efforts in formulation science, regulatory guidance, and scalable manufacturing are also essential for advancing AMP therapeutics closer to clinical practice.

In summary, AMPs have the potential to treat AMR pathogens; however, further investigation is required to fully exploit their properties. ML and AI developments offer crucial tools for AMP discovery and implementation. However, further research should investigate AMP mechanisms to develop multidisciplinary self-assembling AMPs for efficient therapeutic applications. Furthermore, several crucial domains of advancement will shape next-generation peptide delivery platforms. Multi-stimulus-sensitive platforms for peptide administration currently remain unexplored, although most studies have focused on antimicrobial and cancer-prevention therapies. Researchers have used these technologies to respond to multiple stimuli under complex biological conditions, enabling precise targeted delivery. Future studies should focus on improving the synthesis processes and increasing their scalability to meet the demands of healthcare treatment.

## CRediT authorship contribution statement

**Raman Krishnamoorthi:** Writing – review & editing, Writing – original draft, Validation, Supervision, Formal analysis, Conceptualization. **Muthuramalingam Kaviyadharshini:** Writing – original draft, Formal analysis, Conceptualization. **Pambayan Ulagan Mahalingam:** Writing – review & editing, Validation, Conceptualization. **Moovendran Srinivash:** Writing – review & editing, Software, Data curation. **Pitchaimuthu Rajkannan:** Software, Formal analysis, Data curation. **Mohan Keerthivsan:** Writing – original draft, Software, Formal analysis. **Paulraj Suganya:** Writing – review & editing, Validation, Formal analysis, Data curation. **Arokia Vijaya Anand Mariadoss:** Writing – review & editing, Visualization, Validation.

## Declaration of competing interest

The authors declare that they have no known competing financial interests or personal relationships that could have appeared to influence the work reported in this paper.
